# Home-based Extended Rehabilitation for Older people (HERO): study protocol for an individually randomised controlled multi-centre trial to determine the clinical and cost-effectiveness of a home-based exercise intervention for older people with frailty as extended rehabilitation following acute illness or injury, including embedded process evaluation

**DOI:** 10.1186/s13063-021-05778-5

**Published:** 2021-11-08

**Authors:** Matthew Prescott, Amanda Lilley-Kelly, Bonnie Cundill, David Clarke, Sian Drake, Amanda J. Farrin, Anne Forster, Madeline Goodwin, Victoria A. Goodwin, Abi J. Hall, Suzanne Hartley, Mike Holland, Claire Hulme, Silviya Nikolova, Catriona Parker, Phil Wright, Friederike Ziegler, Andrew Clegg

**Affiliations:** 1 Academic Unit for Ageing and Stroke Research, Bradford Institute for Health Research, Bradford Teaching Hospitals NHS Foundation Trust, Bradford, BD9 6RJ UK; 2 Clinical Trials Research Unit (CTRU), Leeds Institute of Clinical Trials Research, University of Leeds, Leeds, LS2 9JT UK; 3 Academic Unit for Ageing and Stroke Research, Leeds Institute of Health Science, University of Leeds, based at: Bradford Institute for Health Research, Bradford Teaching Hospitals NHS Foundation Trust, Bradford, BD9 6RJ UK; 4 College of Medicine and Health, University of Exeter, Exeter, EX1 2LU UK; 5 Academic Unit of Health Economics, University of Leeds, Leeds, LS2 9JT UK; 6 Physiotherapy Department, Bradford Teaching Hospitals NHS Foundation Trust, Bradford Royal Infirmary, Bradford, BD9 6RJ UK

**Keywords:** Older people, frailty, Ageing, Randomised controlled trial, Exercise, Physiotherapy, Rehabilitation, Quality of life, Protocol, Process evaluation, Partially nested, Behaviour change, Complex intervention

## Abstract

**Background:**

The majority of older people (> 65 years) in hospital have frailty and are at increased risk of readmission or death following discharge home. In the UK, following acute hospitalisation, around one third of older people with frailty are referred on for rehabilitation, termed ‘intermediate care’ services. Although this rehabilitation can reduce early readmission to hospital (< 30 days), recipients often do not feel ready to leave the service on discharge, suggesting possible incomplete recovery. Limited evidence suggests extended rehabilitation is of benefit in several conditions and there is preliminary evidence that progressive physical exercise can improve mobility and function for older people with frailty, and slow progression to disability. Our aim is to evaluate the effectiveness of the Home-based Older People’s Exercise (HOPE) programme as extended rehabilitation for older people with frailty discharged home from hospital or intermediate care services after acute illness or injury.

**Methods:**

A multi-centre individually randomised controlled trial, to evaluate the clinical and cost-effectiveness of the HOPE programme. This individualised, graded and progressive 24-week exercise programme is delivered by NHS physiotherapy teams to people aged 65 and older with frailty, identified using the Clinical Frailty Scale, following discharge from acute hospitalisation and linked intermediate care rehabilitation pathways. The primary outcome is physical health-related quality of life, measured using the physical component summary score of the modified Short Form 36- item health questionnaire (SF36) at 12 months. Secondary outcomes include self-reported physical and mental health, functional independence, death, hospitalisations, care home admissions. Plans include health economic analyses and an embedded process evaluation.

**Discussion:**

This trial seeks to determine if extended rehabilitation, via the HOPE programme, can improve physical health-related quality of life for older people with frailty following acute hospitalisation. Results will improve awareness of the rehabilitation needs of older people with frailty, and provide evidence on the clinical and cost-effectiveness of the targeted exercise intervention. There is potential for considerable benefit for health and social care services through widespread implementation of trial findings if clinical and cost-effectiveness is demonstrated.

**Trial registration:**

ISRCTN 13927531. Registered on April 19, 2017.

**Supplementary Information:**

The online version contains supplementary material available at 10.1186/s13063-021-05778-5.

## Administrative information

Note: the numbers in curly brackets in this protocol refer to SPIRIT checklist item numbers. The order of the items has been modified to group similar items (see http://www.equator-network.org/reporting-guidelines/spirit-2013-statement-defining-standard-protocol-items-for-clinical-trials/).

**Table:** 

Title {1}	Home-based Extended Rehabilitation for Older people (HERO): Individually randomised controlled multi-centre trial to determine the clinical and cost effectiveness of a home-based exercise intervention for older people with frailty as extended rehabilitation following acute illness or injury, including embedded process evaluation (protocol).
Trial registration {2a and 2b}.	ISRCTN 13927531 (19/04/2017)
Protocol version {3}	Protocol v5.0 27/01/2020
Funding {4}	National Institute for Health Research, Health Technology Assessment Grant: 15/43/07
Author details {5a}	Matthew Prescott^1^ (Matthew.Prescott@bthft.nhs.uk); Amanda Lilley-Kelly^2^ (A.C.Lilley-Kelly@leeds.ac.uk); Bonnie Cundill^2^ (B.E.Cundill@leeds.ac.uk); David Clarke^3^ (D.J.Clarke@leeds.ac.uk); Sian Drake^2^ (medsdraa@leeds.ac.uk); Amanda J Farrin^2^ (A.J.Farrin@leeds.ac.uk); Anne Forster^3^ (A.Forster@leeds.ac.uk); Madeline Goodwin^2^ (M.E.L.Goodwin@leeds.ac.uk); Vicki Goodwin^4^ (V.Goodwin@exeter.ac.uk); Abi Hall^4^ (A.Hall4@exeter.ac.uk); Suzanne Hartley^2^ (S.Hartley@leeds.ac.uk); Mike Holland^2^ (M.Holland@leeds.ac.uk); Claire Hulme^4^ (C.T.Hulme@exeter.ac.uk); Silviya Nikolova^5^ (S.K.Nikolova@leeds.ac.uk); Catriona Parker^2^ (C.A.Parker@leeds.ac.uk); Phil Wright^6^ (Phil.Wright@bthft.nhs.uk); Friederike Ziegler^1^ (Friederike.Ziegler@gmail.com); Andrew Clegg^3^ (A.P.Clegg@leeds.ac.uk). ^1^Academic Unit for Ageing and Stroke Research, Bradford Institute for Health Research, Bradford Teaching Hospitals NHS Foundation Trust, Bradford, BD9 6RJ ^2^Clinical Trials Research Unit (CTRU), Leeds Institute of Clinical Trials Research, University of Leeds, Leeds, LS2 9JT ^3^Academic Unit for Ageing and Stroke Research, Leeds Institute of Health Science, University of Leeds, based at: Bradford Institute for Health Research, Bradford Teaching Hospitals NHS Foundation Trust, Bradford, BD9 6RJ ^4^ College of Medicine and Health, University of Exeter, Exeter, EX1 2 LU ^5^Academic Unit of Health Economics, University of Leeds, Leeds LS2 9JT ^6^ Physiotherapy Department, Bradford Teaching Hospitals NHS Foundation Trust, Bradford Royal Infirmary, Bradford, BD9 6RJ
Name and contact information for the trial sponsor {5b}	Bradford Teaching Hospitals NHS Foundation Trust: Research Management and Support 01274 382575
Role of sponsor and funder {5c}	The Funder has had no role in trial design, beyond setting the research question the trial addresses. As such the trial is designed specifically to address certain aspects of a brief. In designing the trial, the most significant aspect was around the timely implementation of ‘extended rehabilitation’, after the acute/subacute rehabilitation had finished and the individual had been discharged home from the acute or intermediate care setting. Data collection, management, analysis and interpretation will remain independent of the Funder. The Sponsor maintains oversight of trial processes, but is not involved in trial design or delivery processes. The Sponsor will not participate in data analysis or trial reporting processes.

## Introduction

### Background and rationale {6a}

Frailty is an especially problematic expression of population ageing, with profound implications for planning and delivery of health and social care services. Frailty is characterised by reduced biological reserves and increased vulnerability to adverse outcomes including falls, disability, hospitalisation and care home admission [1]. It develops through age-related decline in several physiological systems, which collectively results in a vulnerability to sudden health status changes triggered by relatively minor stressor events. A majority of older people (> 65 years) in UK hospitals have frailty and are at increased risk of readmission or death following discharge home [2, 3].

Sarcopenia (loss of muscle mass and strength) is a core component of frailty [4, 5] and periods of immobility in older age, such as those experienced during an acute illness or injury, can accelerate loss of skeletal muscle function [6]. Furthermore, the inflammatory response commonly associated with acute illness or injury can lead to catabolism of muscle protein for generation of energy and immune proteins, further accelerating loss of muscle mass and strength [1]. This is especially problematic in frailty because accelerated loss of skeletal muscle function can compromise the capability to perform activities of daily living independently (e.g. walking, dressing, toileting), jeopardising successful functioning in the home environment. This may then increase the need for home care or admission to long-term care residence.

In the UK, following hospital admission with acute illness or injury, approximately one third of older people with frailty are provided with a period of rehabilitation [7]. This National Health Service (NHS) rehabilitation is often provided via ‘intermediate care’ (IC), which is a range of community rehabilitation services predominantly for older people with frailty to promote recovery of independence and reduce premature need for long-term care. IC is typically organised in two general forms: bed-based rehabilitation, or home-based rehabilitation [8], provided across a range of settings (e.g. community hospital and hospital at home/community therapy services). National guidelines for both bed-based and home-based IC recommend only a brief contact (2 to 6 weeks) with services [9]. Findings from the 2014 UK National Audit of Intermediate Care [9] identified that many recipients of IC did not feel ready to leave the service, indicating the possibility of incomplete recovery. Although reduced early readmission to hospital (< 30 days) has been reported in five studies of IC [10], no difference in re-admissions between 60 days and 6 months has been identified, indicating that the early benefits of IC may not be sustained [11]. Indeed, the benefits of rehabilitation in IC are shown to attenuate over time [12]. A key challenge is how to successfully reduce the loss of independence that often affects older people with frailty following hospital discharge. An intervention augmenting usual NHS rehabilitation, provided post-discharge for older people admitted to hospital, is required to improve longer-term outcomes. A programme of progressive physical exercise with integrated behaviour change techniques is a candidate intervention [13].

Exercise has positive physiological effects on skeletal muscle, brain and endocrine systems [1]. Additionally, observational studies have identified a consistent inverse dose-response relationship between physical activity and inflammation [14], which may be especially relevant following acute illness or injury. Randomised controlled trial evidence indicates that exercise can down-regulate inflammation in older people and that the benefit is most pronounced in older people at greatest risk of disability and loss of independence [15]. Systematic reviews of exercise interventions for older people with frailty have reported evidence for improvements in mobility and activities of daily living, but few studies used well-validated frailty tools or measured effects on quality of life and no studies reported on cost-effectiveness [13, 16–18]. Exercise programmes based on progressive strength training were important for functional improvement. This evidence for positive physiological, mobility and functional benefits of exercise in frailty supports our proposal for a home-based exercise intervention to extend the rehabilitation period for older people with frailty following acute illness or injury.

Successful exercise programmes often incorporate strategies to promote behaviour change. Behaviour change strategies demonstrated to have a positive effect on health behaviour adherence include goal setting, self-monitoring, demonstration of behaviour, provision of feedback, use of materials such as exercise logs and manuals, enablement through social support, and extended periods of contact/support from a health care provider [19–22]. It is difficult to draw causal links between single behaviour change strategies and effective health behaviour interventions due to the nuanced and multifaceted nature of many health interventions and health care provider interactions. However, a successful exercise programme supported by physiotherapists will likely need to include many of these behaviour change strategies as elements of the programme and its delivery.

We have developed and tested in a pilot randomised controlled trial (RCT), a home-based exercise intervention for older people with frailty (Home-based Older People’s Exercise (HOPE) programme) [23] aimed at improving strength, endurance and balance. The HOPE programme development has been previously described [23], utilising a staged approach to the synthesis of evidence and a co-design approach and refinement process. The intervention is presented to participants in an exercise manual and delivered by community-based physiotherapy teams. The manualised nature of the intervention and use of face-to-face and telephone support is consistent with evidence-based strategies to promote physical activity behaviour change, and intervention adherence [19, 20]. The HOPE programme has been tested in an earlier pilot trial involving 84 community-dwelling older people, with evidence for feasibility, acceptability, and potential for a positive, clinically important intervention effect on mobility. Informed by previously effective behaviour change programmes, the HOPE programme has been extended by a further 12 weeks of telephone-based support for intervention sustainability for the HERO trial [20].

Here we report the protocol for the HERO trial, a multi-centre individually randomised controlled trial to evaluate the clinical and cost-effectiveness of the HOPE programme for older people with frailty discharged home from hospital or IC services following admission with acute illness or injury.

### Objectives {7}

The overall aim is to establish whether the HOPE programme plus usual care is a clinically and cost-effective extended rehabilitation programme for older people with frailty discharged home from hospital or from IC services after acute illness or injury, when compared with usual care alone.

The primary objective is to evaluate whether the HOPE programme improves physical health-related quality of life, measured using the Physical Component Summary (PCS) of the Modified Short Form 36-item health questionnaire (SF36) 12 months after randomisation.

Secondary objectives evaluate whether the HOPE programme:

1. Improves physical health at 6 months post-randomisation, as measured by the Physical Component Summary (PCS) of the SF36.
2. Improves mental health at 6 and 12 months post-randomisation, as measured by the Mental Component Summary (MCS) of the SF36.
3. Improves functional independence at 6- and 12-month post-randomisation, as measured by the Nottingham Extended Activities of Daily Living (NEADL) and Barthel index.
4. Reduces hospital readmission rates, care home admission rates, hospitalisation due to falls, mortality and overall health and social care resource use at 6 and 12 months post randomisation.
5. Is cost-effective, as measured by differences in cost of service use between groups and the incremental cost-effectiveness ratios (ICERs) using quality-adjusted life years (QALYs) derived from the EuroQol 5 dimension health questionnaire, 5 level (EQ-5D-5L) at 6 and 2 months.

We will also undertake an embedded process evaluation to understand how the intervention is experienced and understood by providers and recipients (participants and their carers), and to explore organisational implications of embedding and sustaining the intervention in preparation for any wider NHS roll-out.

#### Internal pilot objectives

1. To assess whether the provision and acceptability of the intervention meet the pre-defined progression criteria thresholds, for the proportion of participants receiving their first home visit within 3 weeks and retention of intervention participants respectively.
2. To assess whether trial recruitment and 6-month follow-up rates meet the pre-defined progression criteria thresholds.

### Trial design {8}

A multi-centre, parallel group, individually randomised-controlled trial with internal pilot with clear progression criteria and an embedded process evaluation. The intervention arm has a hierarchical structure, as the intervention will be delivered by therapists such that groups of participants (level 1) will receive the intervention from (are nested within) the same therapist (level 2), introducing clustering in the intervention arm only. No clustering occurs in the control arm. Hence this trial is an example of a two-level, partially nested hierarchical design.

## Methods: participants, interventions and outcomes

### Study setting {9}

The study aims to recruit older people with frailty on discharge from general/elderly care, trauma and orthopaedics wards or IC services (bed-based and home-based) after an acute admission to hospital in fifteen NHS hospital trusts within two geographical areas (Yorkshire and South West England). Discharge will be defined as returning home, and the cessation of rehabilitation following the acute admission. Study sites are listed in Acknowledgements.

### Site identification and eligibility {10}

Clinical and research leads for older people and therapy services at English NHS Trust sites in Yorkshire & Humber and the South West will be approached by the HERO trial team and assessed for eligibility. A feasibility questionnaire will determine if sites have services appropriate to support recruitment, delivery of the intervention, and support for trial-related research activity (e.g. data collection). Eligible sites include those with elderly medicine/trauma and orthopaedics services provided at the acute hospital site, availability of bed-based and home-based IC services that routinely receive the trial population transferred from the acute hospital site, agreement by therapy services manager that site recruitment targets are feasible and acceptable for the service to support intervention delivery. Sites already providing routine extended rehabilitation service (> 6 weeks) for older people with frailty following discharge home from hospital or IC will be excluded.

#### Participant identification and eligibility

Participant recruitment will vary by site dependent upon service infrastructure and usual NHS rehabilitation patient pathways. These will be established during site set-up and strategies will be put in place to maximise identification and recruitment of potential patients. Patients will be screened on admission to the relevant ward/service by local research staff. Those meeting the following eligibility criteria will be identified through discussions with ward staff:

- Age ≥65 years.
- Admitted to general medicine/elderly medicine or trauma and orthopaedics care following acute illness or injury then discharged home from hospital or from IC.
- Have mild, moderate or severe frailty, defined as a score of five to seven on the nine-item Clinical Frailty Scale (CFS).
- Ability to complete the Timed Up and Go Test (TUGT) without additional external support.
- Ability to give informed consent to participate in the study.

The following patients will be excluded:

- Permanent care home residents.
- Those with significant cognitive impairment at baseline (defined as Montreal Cognitive Assessment (MoCA) test < 20).
- Recent (< 3 months pre-randomisation) myocardial infarction, or unstable angina.
- Very severe frailty (defined as score of eight on CFS).
- Terminally ill (defined as score of nine on CFS).
- Receiving palliative care.
- Referral at discharge for condition-specific rehabilitation (e.g. pulmonary rehabilitation, stroke rehabilitation, falls prevention programme).
- Another household member in the study.
- Currently participating in the HERO trial or another contraindicated study.

Documented reasons for ineligibility or declining participation will be closely monitored by the Research team as part of a regular review of recruitment progress, to allow for generalisation of study results in accordance with CONSORT reporting guidelines, and to highlight any issues in the identification or recruitment of patients during the internal pilot.

### Who will take informed consent? {26a}

Local research staff will monitor potential participants throughout their admission and seek informed consent approximately 48 h prior to discharge home from hospital or from IC. Eligible participants will be approached and the study will be introduced to them verbally. During this approach, the researchers will also carry out an assessment of capacity to provide informed consent to participate. Interested participants will be given verbal and written information about the trial, given appropriate time to consider involvement and discuss participation with family/friends. Written informed consent will be sought; this will be timed with appropriate proximity to planned discharge. If the potential participant has capacity and chooses to consent, final eligibility assessments will be undertaken (CFS, MoCA, TUGT) and, if appropriate, the baseline assessments will be completed ahead of randomising the participant to their treatment allocation.

#### Eligibility assessments

- Timed Up and Go Test (TUGT) [24]: measures time (seconds) to stand from a chair, walk 3 metres, turn, and return and sit down on the chair. Developed as a basic mobility test for older people [24], the original TUGT validation study identified that those who complete the test in ≥30 s are likely to require assistance with walking, climbing the stairs and leaving the house.
- Clinical Frailty Scale (CFS) [25, 26]: a well-established ordinal (nine point) measure of frailty that has been validated for use in the hospital setting, with higher scores indicating more severe frailty [25, 26]. Individual categories range from very fit (category 1), to terminal illness (category 9). The CFS is a simple tool that clinical and research staff can routinely use to categorise frailty based on an older person’s pre-admission health. This is in contrast with most performance-based frailty measures (e.g. gait speed, grip strength), which can conflate illness severity or sudden changes in mobility with frailty, so are unsuitable in the context of acute illness or injury.
- Montreal Cognitive Assessment (MoCA) [27]: a rapid (≈10 min) screening instrument for mild cognitive dysfunction [27] assessing a range of cognitive domains: a score of 26 or above (from possible 30) is considered normal, scores < 20 indicate the presence of significant cognitive impairment [28].

#### Changes in capacity

If a member of the research team has concerns regarding capacity to provide ongoing consent to participate, a home visit will be scheduled to establish capacity, willingness to continue participation supported by appropriate consent, and support ongoing data collection. This will include seeking advice from a Personal Consultee regarding continued participation in the study. If changes in capacity are noted during intervention delivery the therapist will determine the participant’s willingness and ability to continue with the intervention, in accordance with routine clinical judgements. If the participant is no longer willing to continue with the intervention, this will be documented as a withdrawal from treatment, with involvement in additional activities and data collection to be reviewed by the researcher at follow-up.

### Additional consent provisions for collection and use of participant data and biological specimens {26b}

If the consenting participant has a carer, consent for carer participation in process evaluation activity will be sought. A carer is defined as anyone who cares, unpaid, for a friend or family members who due to illness, disability or a mental health problem cannot cope without their support. Carers who are anticipated to provide support following the participant’s discharge from hospital and are available to support HOPE programme sessions if the participant is randomly allocated to the intervention will be eligible. The local researchers will approach an identified carer once their linked trial participant’s consent has been gained and eligibility has been confirmed.

Participant consent for 3rd party access to anonymised trial data, for purposes of further medical research, is included in the original consent.

## Interventions

### Explanation for the choice of comparators {6b}

Usual care is defined as ‘the wide range of care that is provided in a community whether it is adequate or not, without a normative judgment ’[29].

To increase external validity and relevance of study findings to clinical practice, the study protocol does not restrict access to usual care, in line with our pragmatic study design [30] and the possibility for heterogeneity of usual care treatments available for older people with frailty. For example, usual care at a personal level will depend on individual frailty, level of independence and social predicaments. It is likely to include GP care, district nurse input, and home care packages, but usual care may also include the use of voluntary sector services, day centres, and respite care. Use of and referral to services (including other rehabilitation) will be recorded at baseline and follow-up assessments in both intervention and control groups.

### Intervention description {11a}

A Template for Intervention Description and Replication (TIDieR) checklist [31] is presented in Table 1. 0 in Appendix 1.

### Criteria for discontinuing or modifying allocated interventions {11b}

The intervention therapist will employ flexibility to enable participants to progress or regress, within the programme in line with their health and abilities at a given time, guided by the therapist. If at any stage the participant expresses a wish to pause the HOPE programme, for example due to transient ill health, then that will be accommodated.

### Strategies to improve adherence to interventions {11c}

The therapist will use behaviour change strategies appropriate to each participant to improve adherence to the intervention. These include, but are not limited to, individual goal setting, grading of programme, promote self-monitoring (exercise diary). Therapists will complete a therapy record for each participant documenting implementation of the intervention, any deviations, and the reasons for these deviations.

### Relevant concomitant care permitted or prohibited during the trial {11d}

The study protocol does not restrict access/referral to usual care services for any participants. Additional interventions during the study period will be documented as part of the usual care review. Should the intervention therapist become aware of other new referrals to services, the nature of that referral will be established. The HOPE programme may be discontinued or paused if there is a contraindication to the participant receiving the HOPE programme and other services concurrently.

### Provisions for post-trial care {30}

All participants, when in contact with researchers for purposes of data collection, or therapists for purposes of intervention delivery, may at some stage highlight an unmet care need. The researcher or therapist will then signpost the participant appropriately. Additional therapy outside the HOPE programme will not be provided by the intervention therapists, nor is any specific aftercare planned as part of the trial.

### Outcomes {12}

Outcome data will be collected using self-report postal questionnaires at 6 and 12 months to reduce the risk of detection bias, by telephone assessment if physical disability prevents written communication, or by face-to-face assessment for participants with early signs of cognitive impairment who live alone, by a researcher with experience of working and communicating with an older population. Telephone and face-to-face follow-up assessments will be performed blinded to treatment allocation, where possible, for study participants requiring these methods.

#### Primary outcome

The effectiveness of the HOPE programme will be determined by the SF36 physical component summary (PCS) score measured at 12 months post randomisation. The eight individual scales that comprise the SF36 incorporate the aspects of health and well-being that are relevant for quality of life in older age [32].

#### Secondary outcomes

Secondary outcomes measured at baseline, 6 and 12 months post randomisation (unless stated otherwise), are listed below:

- PCS score of SF36 measured at 6 months.
- Mental component summary (MCS) score of SF36: alongside mental health, this incorporates elements of vitality and social functioning. It therefore has face validity for capturing additional potential benefits of rehabilitation.
- Barthel Index of activities of daily living [33] assesses functional status on a 20 point scale by recording ability to complete ten basic activities of daily living, including bathing, dressing, toileting, mobility and stairs.
- Nottingham Extended Activities of Daily Living (NEADL) [34] measures help needed with instrumental activities of daily living, including walking around outside, doing the housework, using the telephone.
- EuroQol5-Dimension Health Questionnaire (5 levels) (EQ-5D-5L) [35]: measures health-related quality of life, comprising five dimensions: mobility, self-care, usual activities, pain/discomfort and anxiety/depression. Scores are combined and converted into a summary health utility index (0 for dead, 1 for perfect health and negative values for states worse than death).
- Hospital readmission rates, mortality and hospitalisation due to falls, collected using routine Hospital Episode Statistics (HES) and linked Office for National Statistics (ONS) mortality data.
- Care home admission status, recorded by local research staff, who review address details ahead of postal follow-up, to identify those participants admitted to a care home.
- Healthcare Resource Use. In addition to routine HES data, we will use an adapted version of the Health Resource Use data collection form developed for the NIHR Prevention of Falls Injury Trial (PreFit; https://www.journalslibrary.nihr.ac.uk/projects/081441/#/). The form will include health, informal care, social care and voluntary sector resource.
- Falls questionnaire: participants will record number of falls in the prior 6 months, and any broken bones that resulted.
- Twelve-month usual care review: data collected by site research teams from electronic databases at sites regarding secondary and, if available, primary care service use for participants in the 12 months since randomisation.

### Participant timeline {13}

**Fig. 1:**
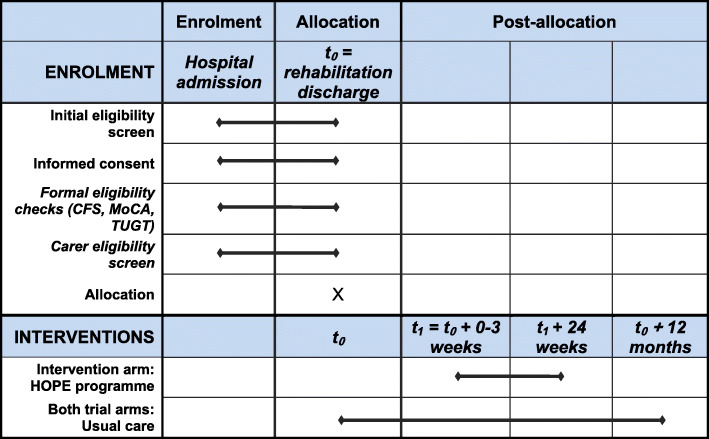
Schedule of enrolment and interventions

**Fig. 2:**
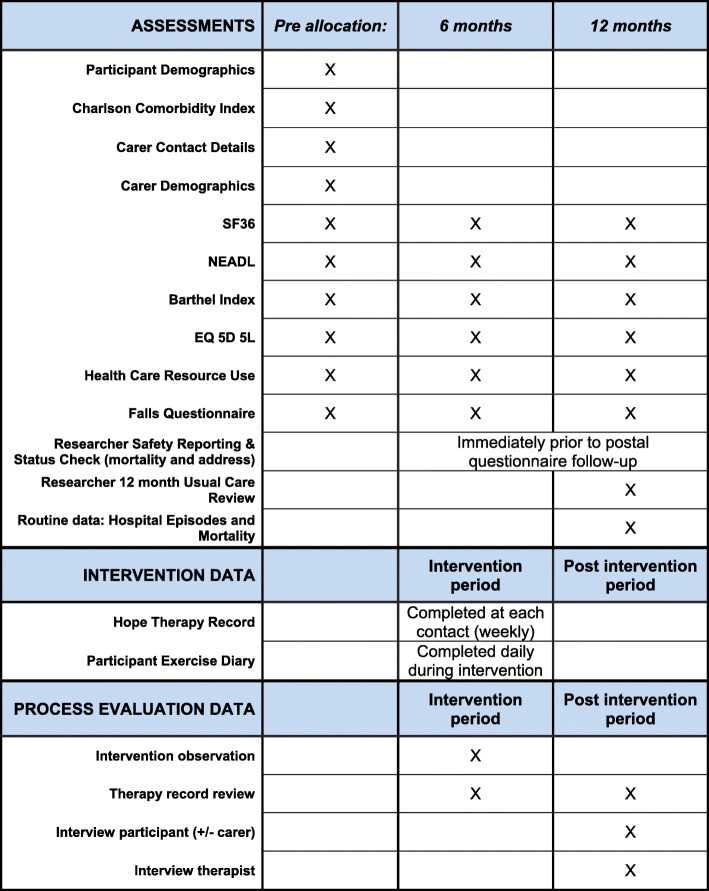
Schedule of assessments

The study timeline for participants is presented in Fig. 1, outlining the schedule of enrolment and intervention and Fig. 2, outlining assessment activities.

### Sample size {14}

A sample of 742 participants (325 control, 417 intervention) will provide 90% power to detect a minimum clinically important difference of 3 points in the PCS, at the 2-sided 5% significance level. The calculations assume a mean PCS score of 30 (SD 9,47) [36] and account for 35% loss to follow-up, clustering in the intervention arm only, average cluster size 7 participants per therapist, an interclass correlation coefficient of 0.03 [37, 38], and a coefficient of variation in cluster size of 0.7 to account for a varying number of participants per therapist.

### Recruitment {15}

Following admission to hospital with acute illness or injury it is anticipated that around one third of frail older people will be discharged directly home from hospital, one third will transfer to IC for a short period of rehabilitation, and one third will be admitted from or discharged to a care home, so will be ineligible, or will have died following admission. On the basis of a 30% recruitment rate, estimates of hospital frailty prevalence, national audit of intermediate care data and NHS secondary care data, and informed by pilot work in which 84 participants were recruited over a 16-month period in a single-site (approximately five per month) whilst ensuring feasibility of intervention delivery by NHS therapy staff as part of routine care [39], we anticipate that recruitment estimates of 4–5 participants at each site each month are achievable, realistic, and feasible for intervention delivery across participating sites considering therapy capacity.

## Assignment of interventions: allocation

### Sequence generation {16a}

Eligible and consenting participants will be individually randomised with a 1.28:1 allocation ratio (HOPE + usual care: usual care) following baseline data collection and within 7 days post discharge. The higher allocation ratio of the intervention arm accounts for a greater level of correlation anticipated in the outcomes for those receiving the HOPE programme, as a result of participants treated by the same community therapy staff.

The method of allocation will use a computer-generated minimisation programme, incorporating a random element, minimised by: site, discharge setting (hospital, bed-based IC, home-based IC), intended level of HOPE programme (level one, two or three) based upon TUGT, and reason for admission (acute illness or injury).

### Concealment mechanism {16b}

Randomisation will be performed by an automated 24-h randomisation service, operated by the CTRU via web address and telephone, and accessible to trained researchers with an authorisation code and PIN.

### Implementation {16c}

Once a participant has consented, the researcher will access a 24-h randomisation service. Following successful randomisation, the researcher and site principal investigator will receive an automated email confirmation from the randomisation service that randomisation was successful, omitting details regarding allocation. Participants will be contacted via letter to confirm their trial allocation, and outline trial procedures that will follow.

Following randomisation, therapy service managers will be notified of participant recruitment via automated email outlining details of participants randomised to the intervention along with discharge date to enable referral to a community therapist and intervention initiation. A list of control participants will be maintained to manage the risk of contamination from therapists delivering the HOPE programme also delivering community services to those in usual care.

## Assignment of interventions: blinding

### Who will be blinded {17a}

Owing to the nature of the intervention, it will not be possible to blind participants or those involved in the delivery of the intervention. The process evaluation will assess the possibility of changed participant/clinical behaviour as a result of this knowledge, and any unanticipated effects.

General practices will be notified of participants’ enrolment on to the study; however, they will not be informed of their allocation status to prevent clinical behaviour change. Other health and social care teams will not be informed about study participation or allocation status.

Those responsible for data collection will be blind to allocation. In the event a researcher becomes unblinded, this will be reported to the CTRU and, where feasible, subsequent follow-up assessments will be completed by an alternative researcher. Data analysts will not be blinded but a detailed statistical analysis plan will be drafted in accordance with CTRU standard operating procedures and will be finalised and agreed by the appropriate members of the research team and independent trial steering committee before any analyses are undertaken.

### Procedure for unblinding if needed {17b}

Trial participation will be recorded in participants’ hospital notes and with their GP. This will not reveal allocation, but will detail where further information can be gained if required. We do not envisage a situation where allocation needs to be revealed outside of regular office hours.

## Data collection and management

### Plans for assessment and collection of outcomes {18a}

The schedule for participant assessments is summarised in Fig. 1: Schedule of enrolment, interventions, and assessments.

Baseline data for all eligible and consenting participants will be collected by the local researcher in the 48 h prior to discharge. These data will be collected directly from the participant, supplemented where appropriate by the team providing care/rehabilitation, and associated records. If discharge is delayed, assessments will be reviewed and repeated where applicable. In addition to outcomes data, the following data will also be collected at baseline:

- Charlson Comorbidity Index [40]: a validated measure used to combine the risk from age, and the risk from comorbid disease into a single variable estimating the risk of death. Higher scores indicate greater comorbidity burden.
- Demographic/descriptive data includes: initials, date of birth, gender, NHS number, ethnicity, living arrangements, employment status, details of hospital admission (reason, date of admission and discharge, discharge from setting), any requirement for researcher supported follow-up at 6 and 12 months.
- Carer contact details and demographics: address, telephone number, age, gender, relationship to participant, care responsibilities.

At 6- and 12-month follow-up, if postal questionnaires are not received back at the CTRU after 3 weeks, a reminder letter and second questionnaire will be posted. If data is not received after two more weeks, local researchers will be asked to establish contact with the participant to offer support, collecting the data over the telephone or via a face-to-face home visit.

Safety data collection and status check for each participant will be requested from sites by the CTRU at 5 and 11 months post randomisation for each participant. Safety data include hospitalisations due to fall and/or fracture. Status check includes mortality and review of contact details which will inform postal questionnaire request at 6 and 12 months.

Site research teams will collect usual care data on secondary and primary care services used by participants when prompted by the CTRU, after 12-month follow-up participant data collection is complete. This will avoid unblinding, if researcher support is required for the participant follow-up assessment.

An application will be made to NHS Digital for HES and linked ONS mortality data, timed to coincide with end of the participant follow-up, in a single data download. There is scope for triangulation of data collected from participants, sites and HES around health care resource use.

All researchers recruiting trial participants and/or involved in data collection will receive initial and ongoing training in data collection tools and trial processes. After initial training, regular communication will be maintained via newsletters, recruitment updates, researcher update teleconferences, and targeted site communications regarding altered trial process. We will also maintain an up-to-date website with trial protocol, contact details and other material. Data collection forms are available on request at ctru-dataaccess@leeds.ac.uk.

### Plans to promote participant retention and complete follow-up {18b}

Initial letters, reminder letters and engagement cards will be used to maximise data return at all-time points. Participant engagement cards precede follow-up by 1 month. These attractive picture cards include a message from the Chief Investigator thanking participants for their participation in the trial. The message reminds participants of the trial purpose: how their data contributes, and of the imminent data collection time point. Participants will receive an unconditional monetary incentive of a £5 gift voucher at each follow-up. A pen will also be sent out with follow-up assessments at both 6 and 12 months for ease of completion and return. As required, researchers will support follow-up. Participant’s family/friends can also support the completion of the assessment. Primary outcome data will be prioritised at the start of the data collection process, and when appropriate, data collection may be staged over additional days, according to participants’ fatigue and at researchers’ discretion.

Participants will be free to withdraw consent and leave the study at any time without giving reasons and without affecting their care. If a participant withdraws consent to participate, clarification will be sought on whether withdrawal is from, for example, participation in the intervention, questionnaire completion or access to health and social care records. Previously collected pseudo-anonymised data will still be used in the analyses.

Individual assessments will not be carried out where the participant appears reluctant to participate (i.e. no response to telephone contacts). However, in the absence of a withdrawal request, outcome data that do not involve participant contact (e.g. from medical or healthcare records) will continue to be collected in these cases.

### Data management {19}

All case report forms sent or received by the CTRU will be coded with the participant’s study number, initials, date of birth and site code. Data will be held securely on paper and entered on a secure electronic database at CTRU. All relevant Standard Operating Procedures, Guidelines and Work Instructions in relation to data management, processing and analysis of data will be followed. CTRU will provide sites with a file to safely maintain essential study documentation and for the retention of completed case report forms and assessments for the study. Interviews for the process evaluation will be audio recorded and professionally transcribed, with any identifiable information removed. Audio files will be securely transferred in encrypted format and stored at the Academic Unit for Ageing and Stroke Research (AUASR).

### Confidentiality {27}

All information collected during the study will be kept strictly confidential, and handled in accordance with the consent provided, adhering to the Data Protection Act, 2018 [41]. All relevant procedures will be followed for processes of transfer, storage, restricted access and disposal of personal information. Upon study completion, sites will archive all study data until authorisation for confidential destruction is provided by the study sponsor. The Trial Master File and documents held by the CTRU will be archived at a secure facility at the University of Leeds.

## Statistical methods

### Statistical methods for primary and secondary outcomes {20a}

A detailed Statistical Analysis Plan (SAP) outlined in this section will be written and approved by the appropriate members of the research team before any formal analyses are undertaken. Following analysis of the progression criteria from the internal pilot, no formal interim analyses are planned. A single final analysis is planned after study closure, when the full database has been cleaned and locked. Statistical analyses will be according to the ITT principle, and statistical significance will be assessed at the two-sided 5% significance level. The ITT population is defined as analysis according to the randomisation and regardless of compliance with the protocol or withdrawal from the trial.

#### Primary analysis

The primary outcome will be compared between arms using a linear mixed-effects heteroscedastic model to account for clustering of outcomes in the intervention arm due to therapist effects [42]. The model will be adjusted for the stratified design factors, age, sex, baseline measure of the outcome, and participant previous engagement or referral to community rehabilitation services. Corresponding 95% confidence interval and *p* values will be reported as well as the adjusted and unadjusted ICC for the intervention arm. Model diagnostics will be used to check that the underlying assumptions are not violated.

#### Secondary outcomes

Secondary outcomes of SF36 MCS score, Barthel Index, NEADL scores, EQ-5D-5L summary index, and A&E attendance or hospital admissions due to falls will be analysed using the same methods as described for the primary outcome and adjusted for the same stratification and participant level covariates. The analysis of the SF36 MCS score will additionally adjust for the baseline SF36 PCS score.

The binary secondary outcomes of new care home placement, hospital readmission, composite of new care home placement or hospital readmission and mortality will be compared between arms using logistic generalised estimating equations or random intercept models to account for heteroscedasticity [43], adjusted for stratification factors and age.

#### Intervention implementation

Descriptive statistics will be used to summarise quantitative data collected on intervention implementation via therapy records and exercise diaries for all intervention participants. This data will support more detailed analysis of intervention fidelity evaluated as part of the process evaluation.

#### Internal pilot

Descriptive statistics will be used to evaluate progression criteria assessing the level of recruitment for each site, follow-up rates, as well as provision and acceptability of the intervention. This analysis will inform study continuation beyond the internal pilot phase. The progression criteria will be assessed based on a traffic light system of green (go), amber (review) and red (stop), as follows:

##### Provision of intervention (assessed at 6 months after the start of internal pilot recruitment)

Green: ≥80% of intervention participants receiving their first home visit within 3 weeks

Amber: < 80% but ≥65% of intervention participants receiving their first home visit within 3 weeks

Red: < 65% of intervention participants receiving their first home visit within 3 weeks

##### Acceptability of intervention (assessed at 9 months after the start of internal pilot recruitment)

Green: ≥80% retention of intervention participants

Amber: < 80% but ≥65% retention of intervention participants

Red: < 65% retention of intervention participants

##### Recruitment (assessed at 6 months after the start of internal pilot recruitment)

Green: ≥4 patients/month/site (measured in months four to six to allow time for recruitment to stabilise)

Amber: < 4 but ≥2 patients/month/site

Red: <2patients/month/site

##### Six-month follow-up (assessed at 12 months after the start of internal pilot recruitment)

Green: ≥80% completion of the SF-36 physical component summary

Amber: < 80% but ≥65% completion of the SF-36 physical component summary

Red: < 65% completion of the SF-36 physical component summary

If any criteria are graded as amber, a rescue plan will be developed outlining steps to be taken to improve intervention provision, recruitment, retention and/or follow-up (as appropriate), and will be approved by the TSC before submission to the funder. If the progression criteria are failed (red), then the internal pilot will not progress to the definitive study. If the progression criteria are met by the end of the internal pilot then the study will continue and outcome data from participants in the internal pilot will be included in the main study analysis.

### Interim analyses {21b}

No formal interim analyses are planned for outcomes. The descriptive data pertaining to the internal pilot will be compiled based on the first 6 months recruitment and associated participant data.

### Methods for additional analyses (e.g. subgroup analyses) {20b}

There are no sub-group analyses planned.

### Methods in analysis to handle protocol non-adherence and any statistical methods to handle missing data {20c}

Missing outcomes, especially those due to death, may not be missing at random, hence first we will explore and summarise the missing data patterns and reasons for missingness to guide the appropriate analytical strategy. If this work confirms that it is reasonable to assume data are missing at random (MAR), the primary analysis will use multiple imputation to deal with missing data. We will include a wide range of variables in the imputation models, including all variables in the substantive analysis, plus, as far as computationally feasible, all variables predictive of the missing values themselves (including potentially time of death) and all variables influencing the process causing the missing data, even if they are not of interest in the substantive analysis. If the initial work does not confirm MAR, we will explore the use of other more complex methods for the primary analysis taking account of data missing not at random (MNAR), such as pattern mixture modelling. To assess the impact of death on our potential treatment effect, we will then undertake a sensitivity analysis by repeating the primary analysis modelling but exclude those participants who have died. A per protocol analysis or Complier Average Causal Effect Model of the primary outcome will also be conducted based on pre-defined criteria associated with intervention adherence.

### Plans to give access to the full protocol, participant level-data and statistical code {31c}

The HERO trial web page (www.bradfordresearch.nhs.uk/hero/) contains the latest version of the trial protocol. Trial outputs will be published in open access journals. Upon study completion, any party may apply to the Chief Investigator and trial team for access to the full protocol and anonymised participant-level data for academic research purposes. An information governance committee including senior trial methodologists will govern data access.

## Economic evaluation

We will carry out within-trial and long-term cost-effectiveness analyses. Analyses will report differences in cost of service use between groups and the ICERs using quality-adjusted life-years (QALYs) derived from EQ-5D-5 L[35].

The primary within-trial analysis will compare direct costs and 12-month outcomes of participants randomised to the HOPE programme (+ usual care) versus control (usual care alone). The perspective adopted will be that of the NHS and Public Social Services. Secondary analyses will adopt a societal perspective taking account of productivity costs and out-of-pocket expenditures incurred by participants. Data collected at baseline, 6 and 12 months will be utilised to estimate incremental cost-effectiveness ratios comparing the intervention with the control group. Mortality and quality of life (derived from the EQ-5D-5L) over the study period will be used to generate QALYs [44]. Sensitivity analyses will consider key cost drivers and factors that might affect the outcomes measured to explore uncertainty in the conclusions drawn [45]. Utility values derived from the SF36 (SF6D) will be included as a sensitivity analysis.

The long-term cost-effectiveness model will again compare effectiveness of the HOPE programme (+ usual care) versus control (usual care alone), from the perspective of the health and social care providers. The decision analytic cost-effectiveness analysis model will use a lifetime horizon to capture the full impact of any mortality differences on the long-term cost-effectiveness. The model will be a Markov or semi-Markov state model. Transition rates for the model will be estimated from the clinical study data. Where this is not possible (e.g. outcomes of hospital readmission/care home placement), we will follow recommended best practice in identifying and synthesising the best available evidence in the literature.

The primary outcome measure will be the QALY. Utility weights will be taken from the tariff recommended by NICE at the time of analysis. Unit costs will be taken from national databases including NHS reference costs and the PSSRU costs of health and social care. Probabilistic sensitivity analyses will be undertaken using Monte Carlo simulation techniques. The outputs reported from the analysis will be the same as for the within-study analysis.

## Process evaluation

In line with the Medical Research Council guidelines [46] an embedded process evaluation will be undertaken. This will use documentary analysis, non-participant observation and semi-structured interviews to explore and understand the implementation of the intervention, and how it is experienced and understood by providers and recipients.

In addition to main trial participation, trial recruits and their carers will be asked to optionally consent to potential participation in the process evaluation. A purposeful sample of consented individuals will be approached across ten trial sites throughout the intervention delivery phase of the trial. Purposive sampling is planned for all types of data collection associated with the process evaluation. The intention is to sample participants with different levels of frailty, level of intervention, gender and age. We will also sample, through observations, intervention delivery at different time points, including first and last visits by therapists or therapy assistants. Trial intervention therapists and therapy service managers will also be approached to consent to their potential involvement in process evaluation activities. Sampling for therapists will seek to include different levels of qualification and years of experience, different gender as well as different sites and regions. Where possible we will sample therapists who have completed intervention delivery to a minimum of three participants.

Consented carers will be approached for process evaluation involvement in line with the participant purposeful sample. Usual care participants will be sampled similarly and invited for interview. Purposive sampling will also be undertaken as part of the documentary analysis in order to ensure records are reviewed from a range of different therapists and participants.

Researchers undertaking the process evaluation will:

- Observe and monitor intervention therapist training, and their engagement with training.
- Engage in informal discussion with therapy teams in early and late stages of intervention delivery to understand how organisational and professional contexts impact on rehabilitation provision to the participant group.
- Interview therapy service managers to establish what constitutes usual care across trial sites.
- Observe home-based delivery of the HOPE programme in a sample of participants across participating sites and across participants with a range of degrees of frailty. Observations will include the interactions between therapists, participants and family members/carers (where present), and note contextual factors potentially influencing programme delivery, receipt and enactment.
- Interview a purposive sample of intervention delivery staff.
- Interview a purposive sample of intervention participants (including those who did and did not engage with the intervention). Carers will also be invited to participate in the semi-structured interviews where it is apparent they have some involvement in supporting participants with the intervention, and/or usual care.
- Interview a purposive sample of trial participants allocated to usual care arm of the trial. This will establish whether they participated in other programmes post discharge provided by the NHS, social care, voluntary or private sector, which may have included similar structured exercise provision, and whether this was evident in particular regions or sites.
- Evaluate intervention adherence using data from participant exercise diaries and therapy records.

Normalisation Process Theory (NPT) will provide the framework for process evaluation [47]. Quantitative and qualitative data to evaluate intervention fidelity will be analysed using descriptive statistics, and content analysis. Field notes and interview data will be analysed using a thematic approach [48].

## Oversight and monitoring

### Composition of the coordinating centre and trial steering committee {5d}

Study oversight will be provided by the independent Trial Steering Committee (TSC), who will meet every 6 months. This comprises of four professionals within a research field involving our study population, with expertise in our trial methodology (statistics, health economics, process evaluation, rehabilitation trials and elderly medicine). The TSC also include two lay personnel who will bring knowledge and expertise from the study population and service user perspective.

The trial management group (TMG) will include the chief investigator and grant co-applicants, CTRU trial management personnel, AUASR trial management and process evaluation teams, and Academic Unit of Health Economics personnel at both the University of Leeds and the University of Exeter. The TMG will also include two lay personnel who will bring knowledge and expertise from the study population and their carers’ perspective. The TMG meetings will be scheduled on a quarterly basis as a minimum, but monthly in the initial phase of trial setup; responsibilities include the set-up and promotion of the trial, on-going monitoring and management of the trial and interpretation and dissemination of results.

### Composition of the data monitoring committee, its role and reporting structure {21a}

For a study of this nature, a separate Data Monitoring Committee is not required. Rather the TSC will adopt a safety monitoring role, with a sub-committee to review safety issues where necessary.

Safety data (deaths and hospitalisation resulting from falls and/or fractures), collected at 5 and 11 months per participant, will be reported by arm to each TMG and TSC meeting and escalated as appropriate. An annual safety report is produced for REC. As deaths are expected within the study population they will not be subject to expedited reporting to the REC, unless the TSC advises otherwise.

### Adverse event reporting and harms {22}

Adverse Events will be reported by sites if classified as related or unexpected (related to the study intervention). Trial sites will be trained in the timely reporting procedures for Suspected and Unexpected Serious Adverse Reactions/Events. Expected Adverse Events data will be reported by trial sites at 5 and 11 months per participant (including death, and hospitalisation rates due to falls and/or fracture). Participants are asked regarding falls and fracture incidence at 6- and 12-month follow-up. We do not specifically ask participants to describe other experienced harms, other than via opportunities to observe and discuss adherence related issues via the process evaluation activity. Safety data as described above will be reported by arm in the final trial report.

### Frequency and plans for auditing trial conduct {23}

Researchers will notify the CTRU if there is a breach of protocol or Good Clinical Practice principles likely to significantly affect participants’ safety, health and wellbeing, or scientific value of the research.

The CTRU/Sponsor reserve the right to conduct intermittent source data verification on a sample of participants. Source data verification will involve direct access to patient notes at the participating hospitals, and other relevant investigation reports.

### Plans for communicating important protocol amendments to relevant parties (e.g. trial participants, ethical committees) {25}

Protocol amendments will be processed in line with HRA and REC guidelines and processes. Sites and investigators will be notified and agreement/capacity to implement sought.

### Dissemination plans {31a}

The NIHR Yorkshire and Humber Applied Research Collaboration (ARC) Research Implementation Advisory Group (RIAG) will be approached to discuss the trial results and potential dissemination of outputs.

Representatives of NHS England and Public Health England will be on the RIAG, along with NHS and local authority commissioners and providers, facilitating widespread dissemination. We will also involve the National ARC Healthy Ageing, Frailty and Dementia collaboration to disseminate findings nationally.

If the HOPE programme is found to be effective, we intend to develop an implementation pack for commissioners to support implementation. We will also make our intervention materials freely available, including the intervention manual, training package and supporting documentation, if effectiveness is demonstrated. Alongside journal publication, we will also aim to present at conferences nationally and internationally. Profession-specific circulations/forums will also be utilised to disseminate results. The clinical/research teams at trial sites will be provided with a trial results summary. We intend to produce an output specifically for the trial participants and their families, summarising the trial findings in a lay publication for circulation. This will be supplemented with information on our web site and social media.

## Discussion

### Screening processes

Feasibility discussions, setting-up and starting recruitment at trial sites, demonstrate that all trial sites are different in terms of service provision and pathways through the acute hospital, to intermediate care services. The foci of screening and recruitment activity differ between organisations, but a challenge facing all research teams is the numerous pathways from admission to ultimate rehabilitation discharge, and the variability and unpredictable duration of patients’ time under the care of those services. Screening and recruitment is challenging when split across many services and settings between both hospital services and separate bed-based and community-based rehabilitation services. Local research teams’ screening and tracking processes for potential participants are likely to evolve over time as services evolve and pathways potentially change.

### Intervention training

To maximise HOPE programme intervention fidelity, a therapist training package will be delivered to all interventional therapists. Intervention training will include clinical reasoning for the HOPE programme exercises, behaviour change strategies and practical delivery of the programme. Research principles including the importance of avoiding contamination via providing intervention principles to control participants will also be discussed. Supervision of therapy staff will be by usual NHS line management. Details of training provision, including content, attendance, duration, and training providers will be documented. Intervention therapists will have access to training materials to support intervention delivery.

Therapists will be provided with ongoing trial-specific training updates via teleconferences and newsletters. Training refresher sessions will be available for therapists where a gap in intervention provision has occurred, or upon request. New therapists joining the team due to rotations/turnover will be provided with the same training package. Any significant intervention training updates will be communicated directly with all therapists individually via email. The central trial team manager (physiotherapist) will seek site intervention team review meetings and feedback in the early stages of site opening.

### Potential impact of the HERO trial

This trial seeks to determine if the HOPE programme as extended rehabilitation for older people with frailty following acute illness or injury can improve physical health-related quality of life. The HOPE programme will be embedded within existing NHS services and pathways in a wide range of trusts both in terms of geography and size, and the diversity of the populations, to make the trial findings generalisable to a population of older people with frailty. The trial is powered such that robust conclusions may be drawn around clinical and cost-effectiveness of a theory-driven exercise intervention with potential for improved health-related quality of life. The results will improve awareness of the rehabilitation needs of older people with frailty. Findings from the embedded process evaluation will contribute to understanding factors which would facilitate wider implementation of the HOPE programme, these include factors influencing trial implementation, intervention fidelity, and the experiences and perspectives of trial participants and providers. The HERO trial findings will have considerable potential for benefit to older people with frailty and for health and social care services, through the commissioning and delivery of evidence-based rehabilitation services, if effectiveness is demonstrated.

## Trial status

Protocol v5.0 27/01/2020. Recruitment began December 2017 and is expected to conclude August 2021.

## Supplementary Information

**Additional file 1:.** TIDieR Checklist

## Abbreviations

A&E: Accident and Emergency
ARC: Applied Research Collaboration
AUASR: Academic Unit for Ageing and Stroke Research
CFS: Clinical Frailty Scale
CTRU: Clinical Trial Research Unit
DGH: District General Hospital
DMC: Data Monitoring Committee
EQ5D-5 L: EuroQol 5 Domains 5 Levels
GP: General practice
HERO: Home-based Extended Rehabilitation for Older people
HES: Hospital Episode Statistics
HOPE: Home-based Older People’s Exercise
HRA: Health Research Authority
IC: Intermediate care
ICC: Interclass correlation coefficient
ICER: Incremental cost-effectiveness ratio
ID: Identification
ISRCTN: International Standard Randomised Controlled Trials Number
ITT: Intention to treat
MAR: Missing at random
MCS: Mental health Component Summary (of SF36)
MNAR: Missing not at random
MoCA: Montreal Cognitive Assessment
NEADL: Nottingham Extended Activities of Daily Living
NHS: National Health Service
NPT: Normative Process Theory
ONS: Office for National Statistics
PCS: Physical health Component Summary (of SF36)
PI: Principle Investigator
PIS: Patient information sheet
PSSRU: Personal Social Service Research Unit
QALY: Quality-adjusted life year
RCT: Randomised controlled trial
REC: Research Ethics Committee
RIAG: Research Implementation Advisory Group
SF36: Modified Short Form 36 item health questionnaire
SPIRIT: Standard Protocol Items: Recommendations for Interventional Trials
TMG: Trial Management Group
TSC: Trial Steering Committee
TUGT: Timed Up and Go Test
