# Supplementary material for: Home-based Extended Rehabilitation for Older people (HERO): study protocol for an individually randomised controlled multi-centre trial to determine the clinical and cost-effectiveness of a home-based exercise intervention for older people with frailty as extended rehabilitation following acute illness or injury, including embedded process evaluation
Source: Trials. 2021 Nov 8;22:783. doi: 10.1186/s13063-021-05778-5 (PMC8576988; doi:10.1186/s13063-021-05778-5)
Supplement: Supplementary file 1 — Additional file 1:. TIDieR Checklist [file 13063_2021_5778_MOESM1_ESM.docx]

# Appendix 1 – TIDieR Checklist

| Table 1.0: TIDieR Checklist |
| --- |
| Name: The Home-based Older People’s Exercise (HOPE) programme |
| Why (rational, theory, goal):  Frailty, loss of independence and decline in health related quality of life become increasingly prevalent with age. These are negatively impacted by acute hospitalisation, and although periods of rehabilitation can offer some restoration of physical function and health related quality of life, these benefits may not be complete when rehabilitation ends, or sustained thereafter. The evidence for exercise programmes providing a positive impact physiologically, often resulting in improved mobility and function in frailty, and behaviour change strategies positively enhancing such programmes, their adoption and adherence in a frail population, provide a rationale for a home-based exercise programme to extend the rehabilitation period for older people with frailty following hospitalisation with acute illness or injury. The primary goal of the HOPE programme is to improve physical health-related quality of life for older people with frailty after acute illness or injury. Secondary goals include improvements in activities of daily living, mental health, and reduced hospitalisations and care home admission. |
| What materials:   - The HOPE programme manual consists of five sections:  1. Information: education around exercise in older age, likely benefits and an overview of the HOPE programme 2. Safety Tips: exercise environment considerations, general precautions for exercising 3. Good Posture: guidance for maximising a good seated or standing posture for exercising 4. Exercises: level specific exercises, each being named, information provided as to intended benefit of the exercise, bullet point text instructions for how to perform the exercise supported by photographs of a person performing the exercise. 5. Staying on Track: information provided here is aimed to help individuals remain engaged with the exercise programme long term, tips include seeking social support, making exercise fun perhaps by doing it with a friend or with music, setting goals, keeping an exercise diary, setting reminders, and what to do on a ‘bad day’.  - The manual is supplemented by a participant exercise diary, enabling the participant to monitor exercise sessions via a simple tick per session up to three times per day. The participant is also provided with a pen, to complete the diary and make notes, and a fridge magnet as a reminder to perform the exercise prescription. All materials are presented in a freestanding reusable bag, in which the items can be stored. The bag includes the HOPE programme logo as a further memory aid. - The therapy record is provided for therapists to complete, based on a standard record used by therapy staff as part of clinical practice. It includes participant information (e.g. demographic detail, comorbidities, and medications) and individual pages for the therapy staff to record a narrative description of each participant contact (home visit and telephone calls). The therapy record pages are structured to provide some prompting to guide content/discussion at therapy sessions. The therapy record also enables recording of information required to calculate costs for intervention delivery (including travel). |
| What procedures:   - The HOPE programme is a 24-week home-based manualised, graded, progressive exercise intervention aimed at improving strength, endurance and balance, required for basic mobility skills like getting out of bed, standing up from a chair, walking a short distance and getting off the toilet. - The programme is graded in to three levels to account for the spectrum of frailty. The functional exercises require no special equipment, and these will be taught by a HOPE programme trained therapist, such that they can be performed without ongoing professional supervision. - Participants will be allocated to intervention level by: - HOPE Level 1: Participants completing the TUGT in ≥30 seconds, who are more likely to require assistance with walking, climbing the stairs and leaving the house. - HOPE Level 2: Participants completing the TUGT in 20-29 seconds, who demonstrate greater variability in mobility, balance and functional ability. - HOPE Level 3: Participants who complete the TUGT in <20 seconds, who tend to be able to get in and out of a chair more easily and climb stairs. - The programme is to be delivered as extended rehabilitation for trial participants allocated to receive it, upon discharge from their rehabilitation pathways following acute hospitalisation. |
| Who provides:   - Suitably trained and experienced community physiotherapists and therapy assistants, familiar with delivering community rehabilitation programmes to older people. - Site physiotherapists and therapy assistants will receive detailed intervention training in interactive workshops delivered by trial physiotherapists experienced in HOPE programme and community rehabilitation delivery. - Intervention training will include: rationale, theory, and goals of HOPE programme, description of intervention materials and procedures, along with strategies and practical delivery of the programme. - Supervision of therapy staff will be via usual NHS line management, with intervention delivery support/advice available from the central trial team. - Access to training materials will be available to trained therapists, and regular updates and communication with trained therapists will be maintained by the central trial teams. - Communication between trial therapists for sharing of experiences and learning will be facilitated by the central trial team, through regular therapist teleconferences, site update meetings and newsletters. - Therapists delivering the HOPE programme will not treat usual care participants referred to community rehabilitation during the study period where feasible. |
| How and where: mechanism and location of delivery   - The HOPE programme is delivered by the trained therapists via one to one home visits and telephone contacts for ongoing support. - Individual home visits and telephone calls will be scheduled in a flexible manner to fit participant availability. - No specialist rehabilitation equipment is required. |
| When and how much:   - The HOPE programme is delivered over a 24 week schedule including five face-to-face home visits, and nineteen telephone contacts for ongoing support. - The exercise routine will typically take less than 15 minutes to complete, and participants are requested to complete the routine three times per day on five days of the week (as able). - A graded approach will be taken to building up the exercise prescription in line with participants physical capacity. - Progression of the programme includes increased repetition of a given exercise prescription, introduction of additional exercises from the HOPE manual, or progression to the next level within the programme. - Review of exercise performance will occur at each weekly contact, and appropriate adjustment of the exercise prescription made. - Participants document exercise session completion in the exercise diary. |
| Tailoring:   - The initial HOPE programme level is tailored to an individual in line with TUGT at baseline. - Physiotherapists will further tailor the programme on an ongoing weekly basis in line with the physical capacity and health status of the individual participant. |
| Modifications:   - The HOPE programme is a 24 week programme, which includes an additional twelve weeks of telephone support when compared to the originally piloted HOPE programme [New 1]. |
| How well (planned):   - Exercise diary and therapy record will capture data regarding the exercise prescription, exercise performance and exercise session completion. - Therapy contact data are recorded including the personnel performing, date and duration, mode of delivery, content of session. - Process evaluation activity includes review of intervention fidelity. This activity will include non-participant observations and review of the training sessions completed, non-participant observations of implementation of the HOPE programme at home visits and telephone contacts, and participant, carer, therapist and therapy service manager interviews across a purposeful sample. |
